# Radiation therapy for stage IVA uterine cervical cancer: treatment outcomes including prognostic factors and risk of vesicovaginal and rectovaginal fistulas

**DOI:** 10.18632/oncotarget.22836

**Published:** 2017-12-01

**Authors:** Masaharu Hata, Izumi Koike, Etsuko Miyagi, Reiko Numazaki, Mikiko Asai-Sato, Hisashi Kaizu, Yuki Mukai, Shoko Takano, Eiko Ito, Madoka Sugiura, Tomio Inoue

**Affiliations:** ^1^ Division of Radiation Oncology, Department of Oncology, Yokohama City University Graduate School of Medicine, Yokohama, Kanagawa, Japan; ^2^ Department of Radiology, Yokohama City University Graduate School of Medicine, Yokohama, Kanagawa, Japan; ^3^ Division of Gynecologic Oncology, Department of Oncology, Yokohama City University Graduate School of Medicine, Yokohama, Kanagawa, Japan; ^4^ Department of Obstetrics and Gynecology, Yokohama City University Graduate School of Medicine, Yokohama, Kanagawa, Japan; ^5^ Department of Obstetrics and Gynecology, Yokohama Minami Kyousai Hospital, Yokohama, Kanagawa, Japan

**Keywords:** cervical cancer, radiation therapy, stage IVA, uterine cervix, vesicovaginal fistula

## Abstract

**Purpose:**

To evaluate the safety and efficacy of radiation therapy for stage IVA uterine cervical cancer and to identify an optimal radiation regimen.

**Results:**

Seventeen of the 28 patients developed recurrence after radiation therapy (local recurrence in 10 and distant metastasis in 12). The local control and distant metastasis-free rates at 3 years in all patients were 61% and 49%, respectively. Fourteen patients died after radiation therapy, and all but 2 died of tumor progression. The disease-free, cause-specific, and overall survival rates at 3 years in all patients were 32%, 49%, and 45%, respectively, and the estimated median survival time was 32 months. Tumor size (*P* = 0.007) and involvement in the lower third of vagina (*P* = 0.006) were significant prognostic factors for local control. Older age (*P* = 0.018) and performance status (*P* = 0.020) were significant prognostic factors for distant metastasis. The presence of hydronephrosis was the sole significant prognostic factor for survival (*P* = 0.026). Only 2 patients developed grade 3 late toxicities (vesicovaginal fistula and radiation proctitis, respectively).

**Materials and Methods:**

Twenty-eight patients with stage IVA uterine cervical cancer received radiation therapy. All patients initially received external pelvic irradiation at a median dose of 50.4 Gy in 28 fractions. Twenty patients also received high-dose-rate intracavitary brachytherapy at a median dose of 22 Gy in 4 fractions. These fraction sizes were lower than conventional sizes. The total median dose for all 28 patients was 68.7 Gy.

**Conclusions:**

Radiation therapy is safe and effective for treatment of stage IVA uterine cervical cancer. The reduced radiation dose per fraction may contribute to the prevention of vesicovaginal fistula formation.

## INTRODUCTION

Uterine cervical cancer is the fourth most common malignancy in women worldwide, following breast, colorectal, and lung cancers. An estimated 530,000 new cases and 270,000 deaths occur annually, accounting for 7.5% of all cancer-related deaths in female patients [1, 2]. Around 85% of cases of uterine cervical cancer arise in less-developed countries, where it is the most common malignancy in women, accounting for almost 12% of all female cancers. Although screening for uterine cervical cancer and vaccination against human papilloma virus are becoming widespread, some patients still develop advanced uterine cervical cancer because of delayed detection. At diagnosis, about 3% of patients have stage IVA uterine cervical cancer with spread of the primary tumor into adjacent pelvic organs, such as the bladder and rectum, based on the International Federation of Gynecology and Obstetrics staging system [3, 4].

Patients with stage IVA uterine cervical cancer are inoperable but are candidates for curative radiation therapy. However, these patients have poorer tumor control and a worse prognosis than patients with tumors up to stage IIIB [4]. Few studies have reported the detailed outcomes of radiation therapy for stage IVA uterine cervical cancer, including its prognostic factors and risk of vesicovaginal and rectovaginal fistulas. We therefore retrospectively reviewed patients with stage IVA uterine cervical cancer treated with radiation therapy and herein discuss the treatment results and optimal radiation regimen.

## RESULTS

### Tumor control

In 26 of the 28 patients, the initial response to treatment was disappearance or marked reduction of the primary tumor, and the remaining 2 patients showed no change. The objective response rate was 93%. However, 17 patients developed recurrence at a median time of 10 months (range, 1–52 months) after radiation therapy. Of these 17 patients, 10 had local recurrence in the pelvis within the radiation field (primary tumor progression in 9 and pelvic lymph node metastasis in 2), and 12 had distant metastases to the lung, liver, bone, and lymph nodes (such as the mediastinal and para-aortic lymph nodes situated outside the radiation field).

**Figure 1:**
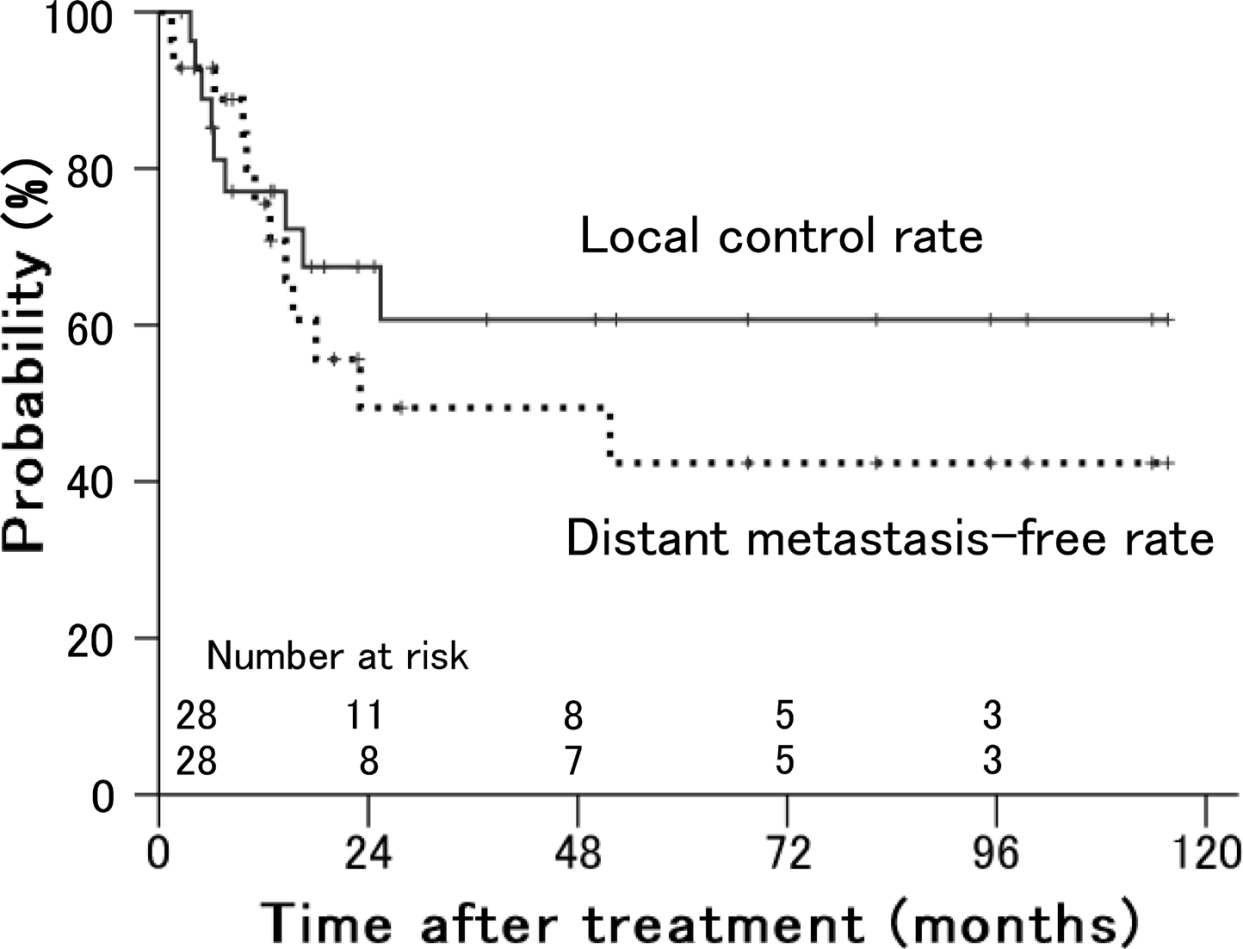
Local control and distant metastasis-free rates in patients with stage IVA uterine cervical cancer treated with radiation therapy

The local control and distant metastasis-free rates in all patients were 61% and 49%, respectively, at 3 years (Figure 1).

### Survival

Fourteen of the 28 patients died at 4–50 months (median, 15 months) after radiation therapy. Of these 14 patients, 12 died of tumor progression and the other 2 of cancer-unrelated causes. There was no mortality associated with treatment. The remaining 14 patients survived during a median follow-up of 45 months (range, 3–116 months) after radiation therapy.

**Figure 2:**
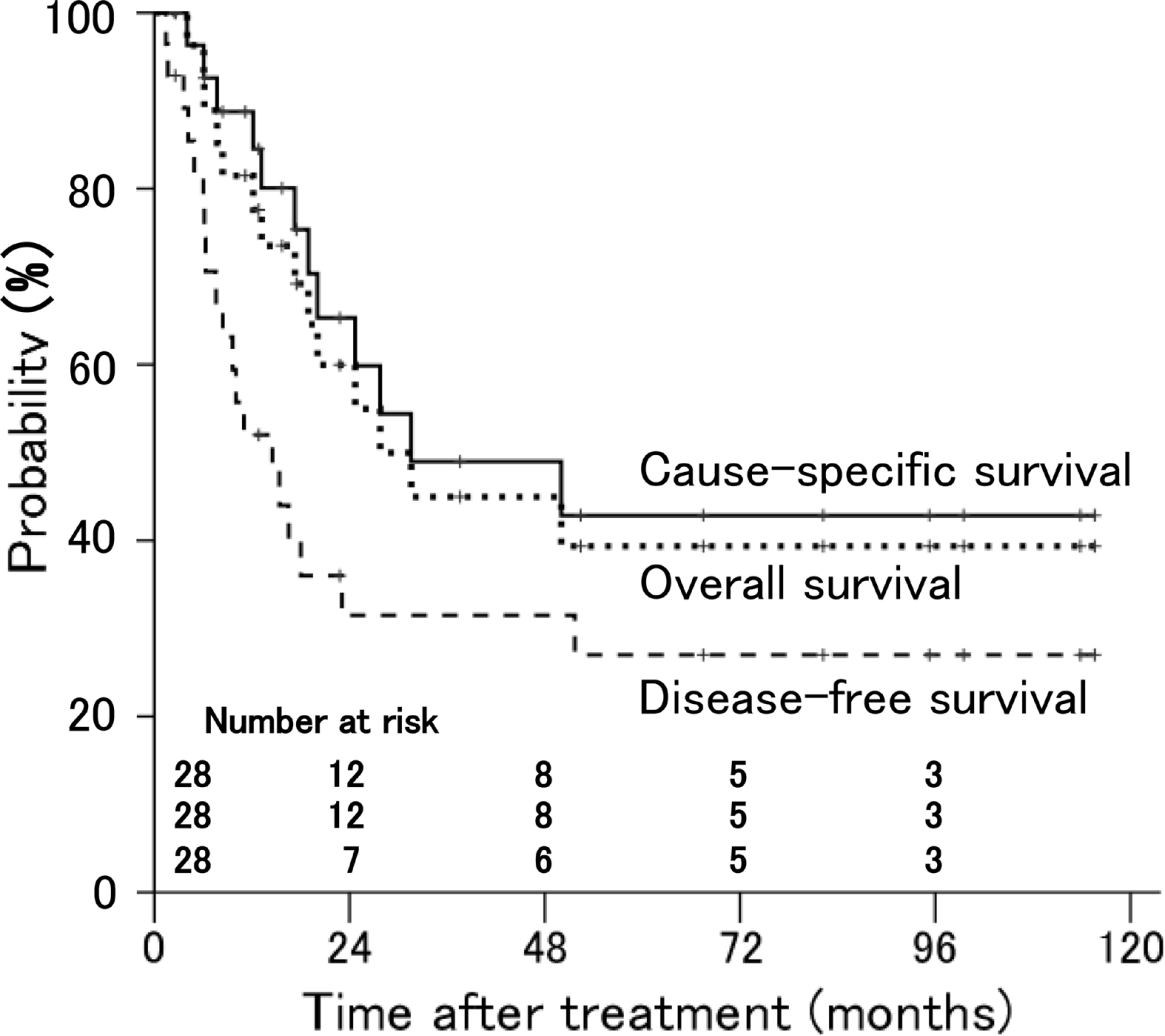
Disease-free, cause-specific, and overall survival rates in patients with stage IVA uterine cervical cancer treated with radiation therapy

The disease-free, cause-specific, and overall survival rates at 3 years in all patients were 32%, 49%, and 45%, respectively, and the estimated median survival time was 32 months (Figure 2).

### Evaluation of prognostic factors

**Figure 3:**
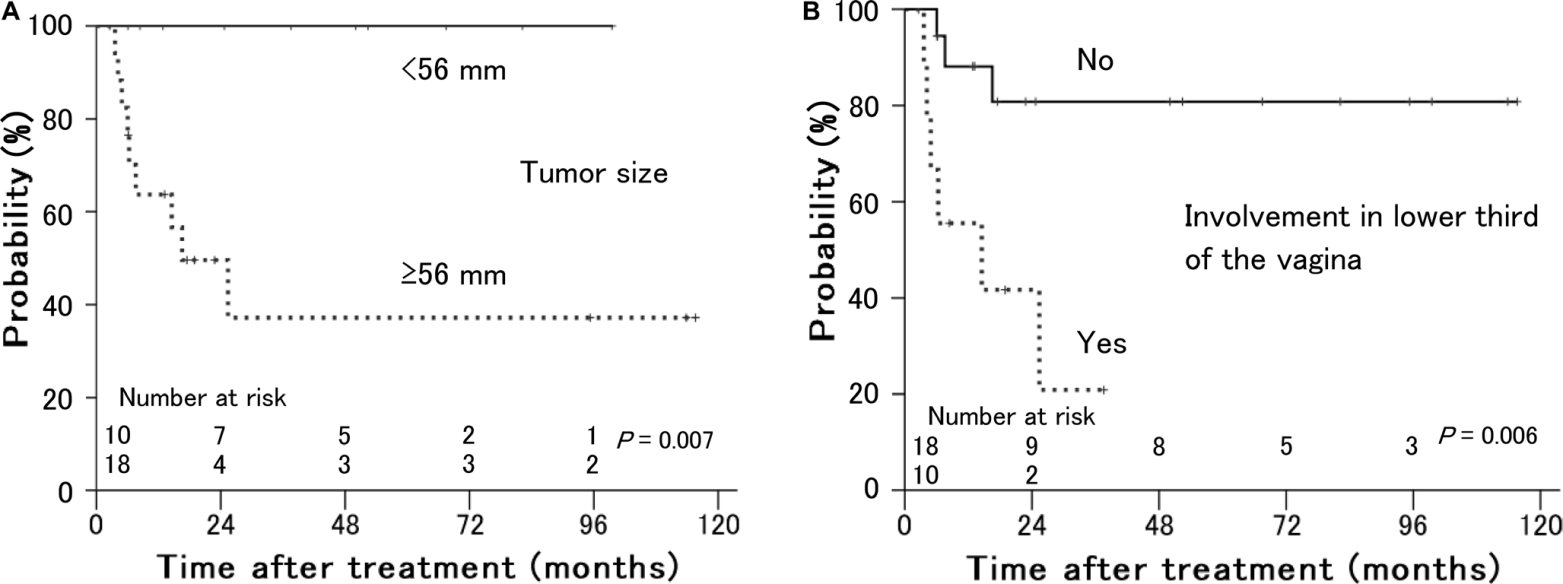
Local control rates in patients with stage IVA uterine cervical cancer treated with radiation therapy according to (**A**) primary tumor size and (**B**) involvement in the lower third of the vagina. Patients with smaller tumors and no involvement in the lower third of the vagina showed better local control.

**Figure 4:**
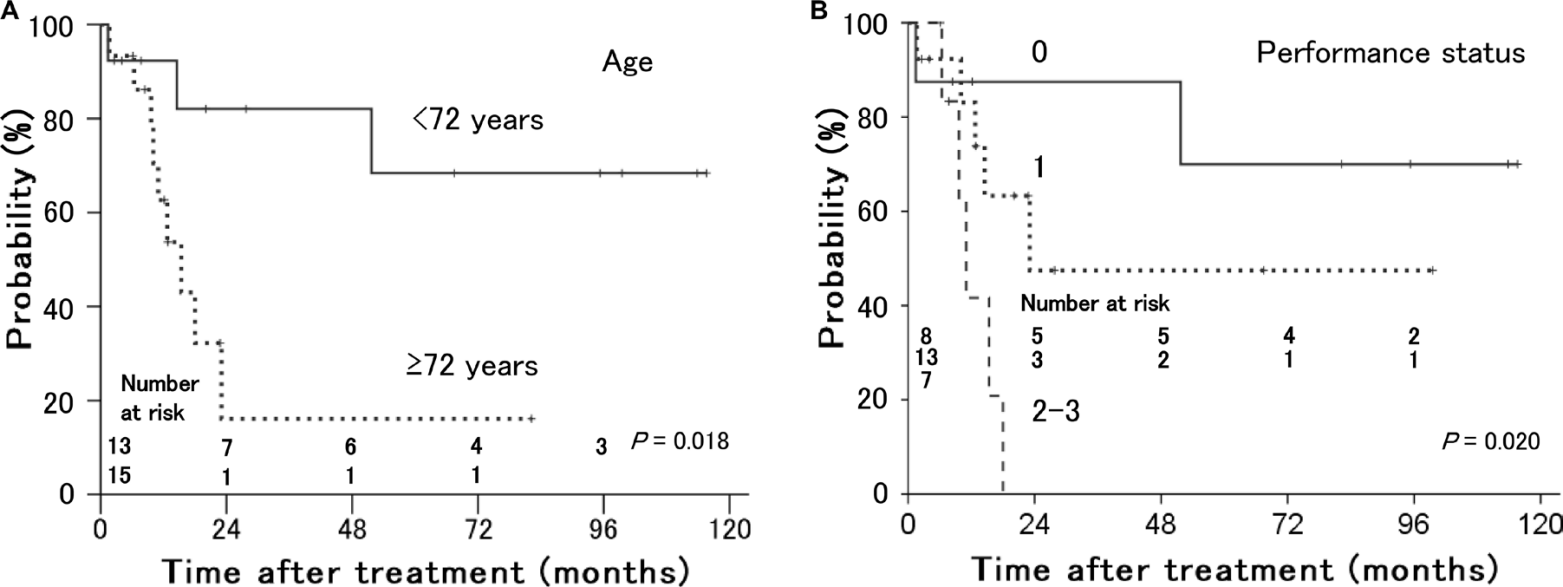
Distant metastasis-free rates in patients with stage IVA uterine cervical cancer treated with radiation therapy according to (**A**) age and (**B**) performance status. Younger patients and patients with a better performance status had a lower incidence of distant metastasis.

**Figure 5:**
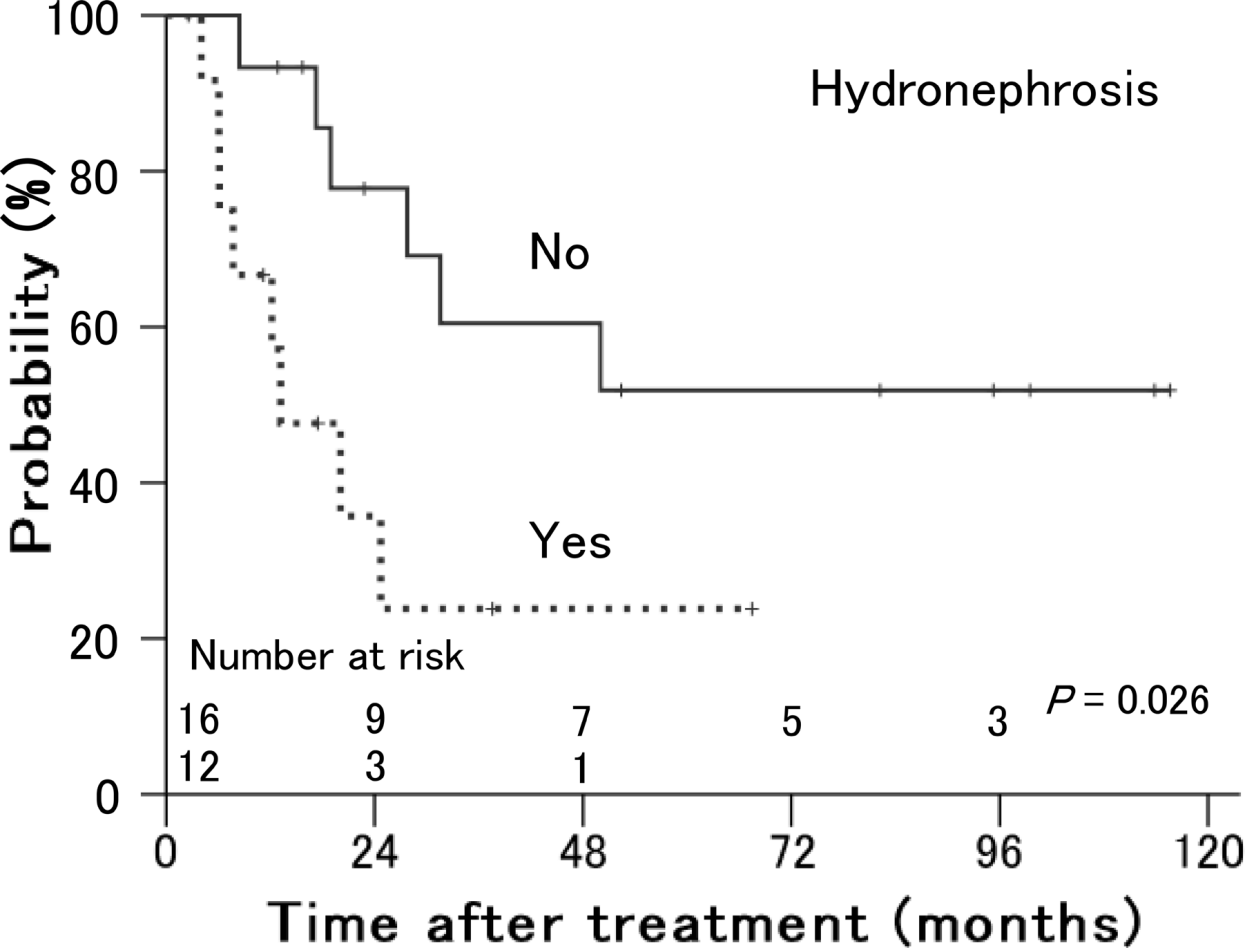
Overall survival rates in patients with stage IVA uterine cervical cancer treated with radiation therapy according to the presence of hydronephrosis Patients without hydronephrosis showed better survival than patients with hydronephrosis.

As shown in Table 1, tumor size (*P* = 0.007) and involvement in the lower third of the vagina (*P* = 0.006) were significant prognostic factors for local control (Figure 3). Of 10 patients with involvement in the lower third of the vagina, 8 had bulky tumors ≥ 56 mm in maximum diameter. Older age (*P* = 0.018) and performance status (*P* = 0.020) were significant prognostic factors for distant failure (Figure 4). The presence of hydronephrosis was considered to be the sole significant prognostic factor for survival (*P* = 0.026, Figure 5).

**Table 1:** Results of Kaplan–Meier and univariate analyses of prognostic factors regarding local control, distant metastasis, and survival

Variables	No. of patients	3-year local control rates (%)	*P* value	3-year distant metastasis-free rates (%)	*P* value	3-year overall survival rates (%)	*P* value
Age (years)							
< 72	13	56	0.494	82	0.018	56	0.246
≥ 72	15	59		16		33	
Performance status							
0	8	88	0.360	88	0.020	63	0.204
1	13	54		48		50	
2–3	7	0		0		0	
Histology							
Squamous cell carcinoma	25	65	0.224	44	0.176	47	0.740
Adenocarcinoma	3	33		100		33	
Primary tumor size (mm)							
< 56	10	100	0.007	54	0.977	69	0.197
≥ 56	18	37		48		28	
Organ infiltration							
Bladder alone	24	65	0.571	47	0.968	45	0.881
Rectum alone or bladder and rectum	4	38		67		38	
Pelvic wall infiltration							
Bilateral	13	45	0.293	25	0.368	26	0.088
Unilateral or none	15	70		60		61	
Involvement in lower third of the vagina							
Yes	10	21	0.006	0	0.228	17	0.092
No	18	81		65		57	
Hydronephrosis							
Yes	12	56	0.127	24	0.072	24	0.026
No	16	68		62		61	
Hydrometra							
Yes	12	75	0.231	56	0.215	63	0.092
No	16	45		42		31	
Pelvic lymph node metastasis							
Yes	12	58	0.540	57	0.592	36	0.935
No	16	60		44		52	
Pretreatment hemoglobin value (g/dL)							
< 11.2	13	64	0.982	43	0.331	34	0.284
≥ 11.2	14	57		50		50	
Pretreatment serum SCC antigen value (ng/mL)^a^							
< 16.8	12	58	0.784	46	0.927	37	0.573
≥ 16.8	12	67		38		50	
Total radiation dose (BED_10_, Gy)							
< 88.0	14	63	0.347	68	0.608	44	0.382
≥ 88.0	14	65		42		50	
Concurrent chemotherapy							
Yes	6	75	0.336	83	0.105	63	0.302
No	22	56		37		40	

Abbreviations: SCC, squamous cell carcinoma; BED_10_, biological effective dose (α/β = 10).

^a^Three patients with adenocarcinoma were excluded from this evaluation.

### Toxicity

There were no grade ≥ 3 acute toxicities except for transient hematological reactions (Table 2). Grade 3 leukopenia, anemia, and thrombocytopenia developed in 2, 3, and 1 patient, respectively. All patients with grade 3 leukopenia and thrombocytopenia were treated with concurrent chemotherapy.

**Table 2:** Number of patients with therapy-related acute toxicities according to the RTOG acute radiation morbidity scoring criteria

Toxicity	Grade			
	1	2	3	4
Hematologic				
Leukopenia	8	4	2	0
Anemia	8	12	3	0
Thrombocytopenia	2	1	1	0
Skin				
Erythema/moist desquamation	3	3	0	0
Upper G.I.				
Abdominal discomfort/ nausea/vomiting	6	3	0	0
Lower G.I.				
Diarrhea	11	1	0	0
Genitourinary				
Frequency of urination/ dysuria/urgency	4	1	0	0

Abbreviations: RTOG, Radiation Therapy Oncology Group; G.I., gastrointestine.

With respect to therapy-related late toxicities, 1 patient developed a grade 3 vesicovaginal fistula and 1 patient developed grade 3 radiation proctitis at 6 and 9 months after treatment, respectively. The patient with the vesicovaginal fistula had bladder invasion and received external irradiation plus intracavitary brachytherapy at a total dose of 131.9 Gy in the biological effective dose (BED_3_, α/β = 3) without chemotherapy. The patient with radiation proctitis received a total dose of 152.6 Gy in BED_3_ without chemotherapy.

## DISCUSSION

Although radiation therapy has been applied in patients with stage IVA uterine cervical cancer, the recurrence rate is high and patient survival is poor. In patients with stage IVA uterine cervical cancer, local recurrence is the most common cause of failure after radiation therapy, but good local control was achieved in the present study. The total median dose of 73.3 Gy in the 2 Gy per fraction-equivalent dose at point A was lower than the dose of ≥ 80 Gy recommended by the American Brachytherapy Society for curative treatment of locally advanced cervical cancer [5]. However, it was close to the standard dose that has been widely used in Japan with good local control [6, 7]. Consequently, the local control rate of 61% at 3 years in the present study was good compared with the rate of 39–59% among patients with stage IVA uterine cervical cancer treated with radiation alone or chemoradiation in previous studies [8, 9].

Many patients with stage IVA disease had bulky tumors in the uterine cervix, with more than half the patients in this study having a cervical tumor of > 60 mm in diameter. Tumor size is an important variable affecting local control in patients at earlier stages. Landoni et al. carried out a randomized controlled trial involving 343 patients with stage IB–IIA uterine cervical cancer treated with surgery or radiation therapy [10]. In 167 patients treated with radiation therapy, patients with tumors of ≥ 4 cm in diameter had a > 2-fold higher local recurrence rate than patients with tumors of < 4 cm (30% vs. 11%, respectively) at median follow-up of 87 months. Similar results have been shown in many previous retrospective studies, and tumor size is an important and useful predictive variable for local control [11–13]. However, few studies have investigated whether tumor size affects local control in radiation therapy for stage IVA uterine cervical cancer alone. In the present study, tumor size was a significant prognostic factor for local control. Tumor size may be significant in stage IVA cancer as well as earlier stage cancer. Some authors have suggested that rectal invasion and the radiation dose to the cervical tumor are prognostic factors for local control, but neither was significant in our study [8, 9]. These outcomes may have been obtained because compared with previous studies, the number of patients with rectal invasion was smaller and almost-curative radiation doses were delivered to more patients in the present study.

Distant metastasis after treatment is also a failure pattern found commonly in patients with stage IIB–IVA cancer, with a high rate of 18–39% [14, 15]. The data on distant failure in only patients with stage IVA cancer are limited, and an accurate rate is not known; however, Fagundes et al. reported an extremely high rate of 75% [16]. In the present study, the distant failure rate reached 51% at 3 years after treatment. The poor prognosis of patients with stage IVA disease results from the high incidence of distant failure as well as poor local control. Some previously published reports, including ours, showed that tumor size was a significant prognostic factor for distant metastasis in patients with stage IB–IVA disease [11, 12, 17]. However, tumor size was not significant in this study. Rather, age and performance status were useful variables for prediction of distant failure. The influence of these variables on survival remains unknown.

Survival of patients with stage IVA uterine cervical cancer is poor, and in previous studies involving patients treated with radiation therapy with or without chemotherapy, the overall survival rates at 3 years were 18–44% [4, 8, 18–20]. Several studies have proved that concurrent use of chemotherapy, particularly platinum-based, with radiation therapy prolonged survival in patients with stage IB–IVA uterine cervical cancer compared with radiation therapy alone [21–23]. Furthermore, the use of consolidation chemotherapy following chemoradiotherapy in an attempt to improve patient survival was recently reported [23, 24]. However, a greater benefit of concurrent chemotherapy was seen in patients at an earlier stage, with 10% improvement in the survival rate at 5 years for patients with stage IB–IIA cancer, 7% for stage IIB, and 3% for stage III–IVA [23]. The benefit of prolonged survival is thus small for patients with stage IVA disease. In addition, intensive treatment can provide enhanced efficacy but may increase the incidence of severe adverse events, such as vesicovaginal and rectovaginal fistulas, in patients with tumor invasion into the bladder and rectum, respectively. In the present study, almost 80% of patients were medically incapable of receiving chemotherapy because of renal dysfunction or old age. However, the overall survival time of 45% at 3 years in our study was more favorable than in previous studies. As a prognostic factor for survival, only the presence of hydronephrosis was significant. Patients with hydronephrosis were likely to develop distant metastasis after treatment, although it was only marginally significant, and this more distant spread may have resulted in poorer survival of patients with hydronephrosis. Previously published data on survival in only patients with stage IVA cancer are also limited. Wakatsuki et al. reported that hydronephrosis was a significant variable for worse survival in patients with stage IVA cancer after definitive radiation therapy: the 2-year disease-free survival rate was 7% in patients with hydronephrosis and 29% in patients without hydronephrosis [8]. The presence of rectal invasion or pelvic lymph node metastasis, the total radiation dose to cervical tumors, and age have also been suggested as predictors of survival by some authors, but none of them was significant in our study [8, 9, 18].

In this study, all therapy-related acute toxicities were temporary and easily manageable. With the exception of hematological reactions, there were no grade ≥ 3 adverse events. All 3 patients with grade 3 leukopenia or thrombocytopenia underwent concurrent chemotherapy. No patients developed grade ≥ 4 acute toxicities. The concurrent use of chemotherapy with radiation therapy in the treatment of cervical cancer has been reported to significantly increase therapy-related acute toxicities, particularly hematological and gastrointestinal reactions [23, 25, 26]. The mild and negligible acute toxicities in the present study probably occurred because many patients were treated with radiation therapy alone without chemotherapy.

Concerning late adverse events, vesicovaginal fistula formation following radiation therapy is uncommon in patients with cervical tumors without bladder invasion. However, it is a major issue in patients with bladder invasion and reportedly has a high incidence after curative radiation therapy. Biewenga et al. treated 20 patients with stage IVA cancer with bladder invasion using external pelvic irradiation at 46.0–50.4 Gy in 1.8–2.0 Gy fractions and brachytherapy at 20 Gy in 20 hours at a medium dose rate or 24 Gy at a high dose rate (24). Some patients also received concurrent chemotherapy with cisplatin and/or hyperthermia. Of these 20 patients, 3 (15%) had vesicovaginal fistulas within 25 months after treatment. Moore et al. reported vesicovaginal fistula formation in 23 patients with stage IVA cancer with bladder invasion [27]. These 23 patients included 7 treated with radiation therapy alone; 14 treated with concurrent chemotherapy with cisplatin, paclitaxel, and/or 5-fluorouracil and radiation therapy; and 2 who received no treatment. External pelvic irradiation with a dose of 50.4 Gy in 28 fractions and low-dose-rate or high-dose-rate (HDR) brachytherapy were used for radiation therapy. Of the 23 patients, 11 (48%) developed vesicovaginal fistulas during a median follow-up period of 19 months. There was no significant difference in vesicovaginal fistula formation between the 2 groups with or without chemotherapy.

In our study, only 1 (3%) of the 26 patients with bladder invasion developed a vesicovaginal fistula. This incidence of vesicovaginal fistula was exceedingly lower than that in the study by Moore et al., in which 11 (48%) of the 23 patients with bladder invasion developed vesicovaginal fistulas. Whether the use of chemotherapy with radiation therapy increases the risk of vesicovaginal fistula formation remains unknown because of poor data. Generally, in the treatment of uterine cervical cancer, chemoradiation increases acute but not late toxicities compared with radiation alone [23, 25, 26]. However, in patients with bladder invasion, vesicovaginal fistula formation can be considered not a therapy-related toxicity but rather the result of effective treatment because of disappearance of the invasive tumor into the bladder wall. Therefore, the rapid reduction in tumor volume that results from intensive treatment may increase the risk of vesicovaginal fistula formation. This suggests that the concurrent use of chemotherapy with radiation therapy should be avoided for patients with bladder invasion. At the same time, however, the absence of chemotherapy may decrease the tumor control and cure rates. Therefore, whether concurrent chemotherapy should be avoided in the treatment of patients with bladder invasion currently remains unclear.

Patients with esophageal cancer characterized by T4 tumors with invasion into an adjacent structure frequently develop esophageal fistulas at a high rate of 10–29% following concurrent chemoradiotherapy [28, 29]. A recent report indicated that in the treatment of patients with T4 esophageal cancer, induction chemotherapy followed by chemoradiotherapy reduced the incidence of esophageal fistula to only 5% [30]. This result cannot be identically applied to patients with bladder invasion of uterine cervical cancer, but slow reduction in the tumor volume can be hypothesized to decrease the risk of fistula formation.

Both fraction sizes used in the present study (1.8 Gy for external pelvic irradiation and 5 Gy for HDR intracavitary brachytherapy) used mostly in the present study were lower than the conventionally and widely used 2 Gy and 6 Gy, respectively. This suggests that a lower radiation dose per fraction may contribute to a decreased incidence of vesicovaginal fistula formation.

The frequency of rectal invasion of uterine cervical cancer is lower than that of bladder invasion; patients with rectal invasion account for only 4–21% of all patients with stage IVA cancer [8, 9, 19, 20, 27]. In our study, only 4 patients (14%) had rectal invasion. Therefore, reports of rectovaginal fistula formation are limited, and the treatment risk remains unknown. Nevertheless, a few studies involving a small number of patients have been performed, and the authors reported that almost none of the patients with rectal invasion developed a rectovaginal fistula following radiation therapy [8, 9, 20, 27]. Similarly, none of the patients in the present study developed a rectovaginal fistula. These results suggest that the incidence of rectovaginal fistula may actually be low. In addition, only 1 patient (3%) developed grade 3 radiation proctitis as a late gastrointestinal adverse event. Grade ≥ 3 gastrointestinal toxicities, including radiation proctitis, occurred in 5–14% of patients with locally advanced cervical cancer treated with radiation therapy in some previous studies [8, 9, 14, 25]. The severe late gastrointestinal toxicity seen in the present study also occurred at a lower incidence than in previous studies.

The present study has some limitations. Selection bias was unavoidable because the study involved a retrospective review. In addition, the number of patients was small and the follow-up period was short because it was a single-institutional study involving only patients with stage IVA uterine cervical cancer, which generally has a poor prognosis. However, considering the fact that very few data on stage IVA cervical cancer are currently available, we believe that the information obtained from this study will be useful for daily medical practice and future prospective studies.

In conclusion, radiation therapy is safe and effective in patients with stage IVA uterine cervical cancer. Uterine cervical cancer is sufficiently curable, even at stage IVA, and aggressive treatment should be considered. However, severe adverse events, such as vesicovaginal fistula formation, are the major issues after treatment. A radiation regimen with a lower dose fraction may decrease the risk of these complications. Further investigations involving larger numbers of patients and longer follow-up periods are required to determine the optimal radiation regimen, with or without chemotherapy, for the treatment of patients with stage IVA uterine cervical cancer.

## MATERIALS AND METHODS

### Patients

From March 1995 to August 2013, a total of 28 patients with stage IVA uterine cervical cancer received radiation therapy with curative intent at our institution. All patients had histopathologically confirmed uterine cervical carcinoma and tumor invasion into the bladder and/or rectum. Bladder and rectal invasion was ascertained by cystoscopy and colonoscopy, respectively. Clinical stage was assessed by chest X-ray and computed tomography (CT), abdominal CT, and pelvic CT and/or magnetic resonance imaging prior to radiation therapy. Of the 28 patients, 12 had pelvic lymph node metastases but none had distant metastasis. Lymph node enlargement of > 10 mm in the short axis on CT was defined as metastasis. Consequently, all patients were clinically diagnosed with stage IVA based on the TNM classification defined by the Union for International Cancer Control [31].

Patient characteristics are shown in Table 3. Informed consent was obtained from all patients before treatment. This study was approved by the institutional review board of our institution.

**Table 3:** Patient and tumor characteristics

Number of patients	28
Age (years)	72 (29–92)
Performance status (ECOG)	
0	8 (29)
1	13 (46)
2	4 (14)
3	3 (11)
Histology	
Squamous cell carcinoma	25 (89)
Adenocarcinoma	3 (11)
Primary tumor size (maximum diameter, mm)	61 (45–110)
Organ infiltration	
Bladder alone	24 (86)
Rectum alone	2 (7)
Bladder and rectum	2 (7)
Parametrial infiltration	
Bilateral	27 (96)
Unilateral	1 (4)
Pelvic wall infiltration	
Bilateral	13 (46)
Unilateral	13 (46)
None	2 (7)
Involvement in the vagina	
Lower third of the vagina	10 (36)
Upper two-thirds of the vagina	15 (54)
None	3 (11)
Hydronephrosis	
Yes	12 (43)
No	16 (57)
Hydrometra	
Yes	12 (43)
No	16 (57)
Pelvic lymph node metastasis	
Yes	12 (43)
No	16 (57)
Pretreatment hemoglobin value (g/dL)	7.6–14.0 (11.2)
Pretreatment serum SCC antigen value (ng/mL)	1.1–156.3 (16.8)
Total radiation dose (BED_10_, Gy)	59.5–105.4 (88.0)
Concurrent chemotherapy	
Yes	6 (21)
No	22 (79)

Data are presented at *n* (%) or median (range).

Abbreviations: ECOG, Eastern Cooperative Oncology Group; SCC, squamous cell carcinoma; BED_10_, biological effective dose (α/β = 10).

### Radiation therapy

All 28 patients initially received external irradiation to the whole pelvis in antero-posterior opposed fields or antero-posterior and bilateral fields (box fields) with 10–15-MV X-rays. Patients received 1.8 Gy per day, 5 times per week, to a total dose of 45.0–50.4 Gy (median, 50.4 Gy) in 25–28 fractions (median, 28 fractions). Twenty of the 28 patients also received HDR intracavitary brachytherapy along with external irradiation to treat the primary cervical tumors. Total doses of 5–29 Gy (median, 22 Gy) in 1–5 once-weekly fractions (median, 4 fractions) of 3.5–6.0 Gy (median, 5 Gy) were delivered to point A using an HDR Ir-192 source [32]. Although intracavitary brachytherapy was considered for the control of primary tumors with higher doses in all patients, it was abandoned in the remaining 8 patients because of dementia, patient refusal or technical difficulties. The primary tumors in these 8 patients were treated with external irradiation alone to total doses of 50.4–59.4 Gy in 28–33 fractions, consisting of whole pelvic irradiation of 45.0–50.4 Gy and a local radiation boost of 0–14.4 Gy. The total doses administered to all 28 patients were thus 50.4–79.4 Gy (median, 68.7 Gy), and the overall treatment time was 44–72 days (median, 51 days). The biological effective dose (BED) was calculated from the total physical dose according to the linear quadratic model using α/β ratios of 10 and 3 for early and late responding tissues, respectively. The median BED_10_ and BED_3_ were 88.0 Gy (range, 59.5–105.4 Gy) and 131.0 Gy (range, 80.6–166.0 Gy), respectively [33]. The BED_10_ was also considered as the dose of radiation that was effective for tumor treatment, and the median dose of 88.0 Gy corresponded to 73.3 Gy (range, 49.6–87.8 Gy) in the 2 Gy per fraction-equivalent dose.

### Chemotherapy

Six of the 28 patients also received concurrent chemotherapy. These patients received 1–5 courses of mainly weekly intravenous cisplatin (40 mg/m^2^) during radiation therapy. For the remaining 22 patients, chemotherapy was unacceptable because of renal dysfunction or old age.

### Follow-up and evaluation criteria

Patients were examined by pelvic CT within 1 month after completion of irradiation, and underwent subsequent follow-up CT scans at 3–12-month intervals. When the irradiated tumors show no progression, they were considered locally controlled.

Acute and late toxicities associated with radiation therapy were evaluated using the Radiation Therapy Oncology Group (RTOG) acute radiation morbidity scoring criteria and the RTOG/European Organization for Research and Treatment of Cancer late radiation morbidity scoring scheme, respectively [34]. Acute and late toxicities were defined as radiation-induced toxicities occurring within and after the first 3 months of beginning radiation therapy, respectively.

### Statistical analysis

Actuarial survival and disease-control rates were calculated from the beginning of radiation therapy, according to the Kaplan–Meier method [35]. The log-rank test was applied to detect probable prognostic factors considered predictable among various patient and tumor factors in the univariate analyses. A *P* value of < 0.05 was considered statistically significant. All statistical analyses were performed using the statistical software IBM SPSS version 22 (IBM, Armonk, NY, USA).

## Abbreviations

BED: Biological effective dose
HDR: high-dose-rate
CT: computed tomography
RTOG: Radiation Therapy Oncology Group
